# Targeting Apoptosis Signaling in Pancreatic Cancer

**DOI:** 10.3390/cancers3010241

**Published:** 2011-01-11

**Authors:** Simone Fulda

**Affiliations:** Institute for Experimental Cancer Research in Pediatrics, Goethe-University Frankfurt, Komturstr. 3a, 60528 Frankfurt, Germany; E-Mail: simone.fulda@kgu.de; Tel.: +49 69 67866557; Fax: +49 69 6786659157

**Keywords:** apoptosis, pancreatic cancer, TRAIL, IAPs, mitochondria

## Abstract

The ability to escape apoptosis or programmed cell death is a hallmark of human cancers, for example pancreatic cancer. This can promote tumorigenesis, since too little cell death by apoptosis disturbs tissue homeostasis. Additionally, defective apoptosis signaling is the underlying cause of failure to respond to current treatment approaches, since therapy-mediated antitumor activity requires the intactness of apoptosis signaling pathways in cancer cells. Thus, the elucidation of defects in the regulation of apoptosis in pancreatic carcinoma can result in the identification of novel targets for therapeutic interference and for exploitation for cancer drug discovery.

## 1. Introduction

Tissue homeostasis critically depends on a subtle balance between cell growth and cell death and is typically disturbed in human cancers [1].This implies that a reduced rate of programmed cell death (apoptosis) and/or an increase in cell proliferation can lead to tumor formation [1]. Apoptosis is a highly conserved form of cell death that already occurs in lower organisms [2]. Apoptosis exerts vital functions in many physiological processes and is found to be deregulated in a large variety of human diseases [1]. For example, a hallmark of human cancers including pancreatic carcinoma is the inability of cells to undergo apoptosis in response to an apoptotic stimulus [3,4]. Deregulated apoptosis programs are often also the underlying cause of primary or acquired resistance of pancreatic carcinoma to current therapies, including chemo-, radio- or immunotherapy, since these treatment strategies primarily act by triggering the intrinsic cell death program in target cancer cells [5]. The elucidation of apoptosis pathways and their deregulation in pancreatic cancer therefore harbors a great potential for the development of novel experimental cancer therapeutics that are targeted to the underlying molecular mechanisms of treatment resistance. Such an approach is expected to have the ability to bypass classical drug resistance mechanisms.

## 2. Pancreatic Cancer

Pancreatic cancer represents currently one of the leading causes of cancer deaths in the Western world with a very poor prognosis despite intensive protocols [6,7]. This poor outcome is, at least in part, due to the resistance of pancreatic cancer to the currently available treatment options and still constitutes one of the most challenging problems in oncology [8]. Evasion of apoptosis substantially contributes to this treatment failure, since cytotoxic cancer therapies depend on the induction of apoptosis in cancer cells in order to be effective [5]. Thus, strategies that target defective apoptosis programs may open new perspectives to improve the prognosis of pancreatic cancer patients.

## 3. Apoptosis Signaling Pathways

A large variety of stimuli that originate both from the exterior of the cell, for example death receptors, chemotherapeutic agents or therapeutic antibodies, or alternatively from the inside of a cell, such as reactive oxygen species or metabolic end products, can initiate apoptosis [1]. In case of caspase-dependent apoptosis, the activation of apoptosis signaling pathways results in the activation of caspases [9]. Caspases are a family of cysteine proteases that act as death effector molecules in many forms of cell death [9]. Caspases are synthesized as inactive proenzymes and cleavage or dimerization is required for their activation [9].

Caspases can become activated by two principal pathways, *i.e.*, the death receptor (extrinsic) pathway and the mitochondrial (intrinsic) pathway [10]. In the death receptor pathway, the engagement of death receptors by their corresponding ligands, for example agonistic TRAIL receptors, leads to the activation of the initiator caspase-8 [11]. Once activated caspase-8 either directly cleaves effector caspases such as caspase-3 or, alternatively, initiates the mitochondrial pathway by cleaving Bid. The processed form of Bid, *i.e.*, tBid, in turn translocates to mitochondrial membranes, where it initiates the release of mitochondrial intermembrane space proteins such as cytochrome c into the cytosol [12,13]. In the cytosol, cytochrome c forms a multimeric complex together with Apaf-1 and caspase-9 termed the apoptosome complex, which is responsible for caspase-3 activation [13].

It is important to note that there are also additional forms of cell death beyond apoptosis [14]. These non-apoptotic cell death modes include e.g. necrosis, autophagy, lysosomal cell death or mitotic catastrophe [14]. There is mounting evidence that the type of cell death is highly dependent on the cellular context and that different forms of cell death may occur in parallel in a given system.

## 4. Targeting Apoptosis for the Treatment of Pancreatic Cancer

### 4.1. Death Receptor Signaling as Therapeutic Target in Pancreatic Cancer

Death receptors are part of the tumor necrosis factor (TNF) receptor gene superfamily of transmembrane receptors that are characterized by an intracellular domain called the “death domain” [11]. This domain transmits the death signal from the surface of a cell to intracellular signaling pathways and serves as a platform for the recruitment of signaling molecules that transmit the death signal such as the adaptor protein FADD and caspase-8 [11]. Binding of death receptor ligands such as TRAIL to their corresponding receptors leads to the recruitment of FADD and caspase-8 to the activated death receptor. This results in the formation of the death-inducing signaling complex (DISC) at the plasma membrane, *i.e.*, which in turn leads to caspase-8 activation [11].

The concept is to target death receptors for cancer therapy, since death receptors can directly engage the cell death program of cancer cells. To this end, TRAIL presents the most promising clinical candidate, since it preferentially triggers apoptosis in cancer over non-malignant cells [11]. The presence of TRAIL receptors exposed on the surface of cell membranes offers the possibility of inducing apoptosis in cancer cells by the exogenous administration of compounds capable of directly engaging two agonistic TRAIL receptors, *i.e.*, TRAIL-R1 and TRAIL-R2 [11]. By comparison, two antagonistic decoy receptors, TRAIL-R3 and TRAIL-R4, bind TRAIL, but do not signal cell death, and osteoprotegerin acts as a soluble decoy receptor [11]. At least one of the agonistic TRAIL receptors has been reported to be expressed in human pancreatic carcinoma tissue, while conflicting reports have been made on expression of TRAIL decoy receptors [15-18]. Although the majority of pancreatic carcinoma cell lines were found to express the essential signaling molecules of the TRAIL pathway [19-21], most of them turned out to be refractory to TRAIL [21,22]. However, sensitivity towards TRAIL could be restored by various combination therapies, e.g. using chemotherapeutics (camptothecin, cisplatin, celecoxib) that downregulate c-FLIP expression [23]. Also in a xenografts mouse model of pancreatic adenocarcinoma, TRAIL together with gemcitabine showed a greater anti-tumor effect than either monotherapy used alone [24]. Along this line, anti-TRAIL receptor 2 antibody combined with CPT-11 caused inhibition of tumor growth in an orthotopic model of pancreatic cancer [25]. Also, pharmacologic or genetic inhibition of CDK4 enhanced the TRAIL sensitivity [26]. Moreover, the estradiol metabolite 2-Methoxyestradiol promoted TRAIL-induced apoptosis by upregulating TRAIL receptors via generation of oxidative stress and JNK activation [27]. Combination therapy with the proteasome inhibitor bortezomib similarly enhanced TRAIL-induced apoptosis via upregulation of TRAIL-R1/TRAIL-R2, downregulation of c-FLIP_L_ and increase in Bak [28]. These various combination strategies indicate that the antitumor activity of TRAIL can substantially be augmented by the addition of other cytotoxic principles.

Expression levels of O-glycosyltransferases may serve as biomarkers to predict TRAIL sensitivity, as they are overexpressed in several cancers and regulate TRAIL-induced apoptosis via modulation of TRAIL-R1 or -R2 [29].

Various agents have been developed in recent years to target the TRAIL system for clinical application [30]. For example, soluble TRAIL and agonistic antibodies directed against the TRAIL receptors TRAIL-R1 and -R2 were developed [31,32]. In pancreatic carcinoma, Apomab (agonistic antibody against TRAIL-R2) was shown to be effective in pancreatic cancer as a single agent and in combination with chemotherapy [33]. At present, TRAIL-R1 monoclonal antibodies are being tested in early clinical trials alone or in combination with chemotherapeutic drugs [34].

In addition to the induction of apoptosis, TRAIL may also exert non-apoptotic functions, including activation of survival cascades such as NF-κB, PI3K/Akt or Ras/Raf/ERK pathways [35]. For example, TRAIL was shown to enhance metastasis formation in an orthotopic mouse model of human pancreatic carcinoma [36]. Thus, clinical protocols with TRAIL have to take also those activities into consideration.

### 4.2. “Inhibitor of Apoptosis” (IAP) Proteins as Therapeutic Targets in Pancreatic Cancer

“Inhibitor of Apoptosis” (IAP) proteins comprise a family of endogenous caspase inhibitors with eight human analogues, *i.e.*, XIAP, cIAP1, cIAP2, survivin, livin (ML-IAP), NAIP, Bruce (apollon) and ILP-2 [37]. All IAP proteins harbor at least one baculovirus IAP repeat (BIR) domain, which presents the interaction domain with caspases. Among the IAP family proteins, XIAP has shown the most potent anti-apoptotic effects by inhibiting active caspase-3 and -7 and by preventing caspase-9 activation [37,38]. The IAP family protein survivin not only regulates apoptosis, but also controls mitotic events [39]. IAP proteins are negatively controlled at several levels, e.g. by mitochondrial (Smac/DIABLO) or nuclear proteins (*i.e.*, XIAP-associated factor 1 (XAF-1)) [37]. IAP proteins are also targets of caspase-mediated cleavage as well as ubiquitination-mediated proteasomal degradation via auto-and heteroubiquitination through the RING domain of IAP proteins [37].

In pancreatic cancer, high expression levels of several of the IAP proteins have been reported in comparison to non-malignant pancreatic ductal cells or pancreatic tissue, including XIAP, cIAP2, survivin and livin [40-43]. In addition, low expression levels of XAF1 were recently shown to correlate with shorter survival times in pancreatic cancer [44]. In multivariate analysis, XAF1 expression turned out as an independent prognostic indicator of the survival of these patients [44].

In order to target the expression and/or function of IAP proteins in pancreatic cancer, a number of approaches were developed. So far, most of these strategies are directed against XIAP, since XIAP possesses the most potent anti-apoptotic properties among the IAP proteins [38], for example, RNA interference (RNAi) or antisense oligonucleotides. To this end, RNAi-mediated downregulation of XIAP was reported to increase apoptosis of pancreatic carcinoma cells following treatment with death receptor ligands, such as TRAIL or agonistic anti-CD95 antibodies, as well as after γ-irradiation and also suppressed colony formation [21,45]. Additionally, the combination of XIAP inhibition and TRAIL even overcame Bcl-2-conferred resistance [46]. This involved a switch of type II cells, which require the mitochondrial contribution to TRAIL-induced apoptosis, to type I cells in which TRAIL triggers apoptosis irrespective of Bcl-2 overexpression [46]. Also, inhibition of XIAP cooperated with TRAIL to trigger regression of established pancreatic carcinoma in a tumor regression model in xenograft-bearing mice [46]. Similarly, loss of XIAP protein upon administration of XIAP antisense oligonucleotides increased TRAIL-mediated apoptosis in a pancreatic carcinoma cell line [47]. Downregulation of XIAP or cIAP2 also increased the response to anticancer drugs including doxorubicin, paclitaxel, gemcitabine and cisplatin, at least in some pancreatic cancer cell lines [41,42]. Loss of XIAP protein upon administration of XIAP antisense oligonucleotides correlated with increased sensitization to TRAIL-mediated apoptosis in a pancreatic carcinoma cell line [47]. XIAP antisense oligonucleotides against XIAP are currently under evaluation in early clinical trials [48].

The binding groove of the BIR3 domain of XIAP, which binds Smac, has served as a target for the design of compounds that inhibit XIAP [49]. In pancreatic cancer, Smac peptides were reported to sensitize pancreatic cancer cells to both death-receptor-or anticancer drug-induced apoptosis [50]. To facilitate intracellular delivery, a carrier was coupled to Smac peptides, for example the protein transduction motif of the HIV Tat protein [50]. Smac mimetics were also found in another study to potentiate the chemotherapy response [51]. In addition, small molecule XIAP inhibitors synergized with TRAIL to induce apoptosis both *in vitro* and *in vivo*, causing regression of pancreatic carcinoma [43]. Also, XIAP inhibitors potentiated radiosensitivity of pancreatic carcinoma cells by enhancing caspase cleavage and subsequently apoptosis in response to γ-irradiation [45].

In addition to synthetic small molecule inhibitors, Embelin, a natural compound from the Japanese Ardisia herb, was identified as a XIAP inhibitor [52]. In pancreatic carcinoma cell lines, Embelin enhanced TRAIL-induced apoptosis [53].

Besides the BIR2 domain of XIAP, small molecule compounds were also designed against the BIR2 domain of the protein. To this end, the screening of a polyphenylurea library resulted in the identification of several non-peptidic compounds with potent binding to the BIR2 domain of XIAP [54,55]. In pancreatic carcinoma cells, these polyphenylurea compounds induced apoptosis as single agents and also increased the induction of apoptosis following treatment with gemcitabine, TRAIL or irradiation [40].

### 4.3. Mitochondria as Therapeutic Target in Pancreatic Cancer

The Bcl-2 family of proteins comprises both anti-apoptotic members, e.g. Bcl-2, Bcl-X_L_, Mcl-1, and pro-apoptotic proteins including Bax, Bak, Bad and BH3 domain only-proteins [12]. Imbalances in the ratio of anti-apoptotic *versus* pro-apoptotic Bcl-2 proteins with a relative increase in the anti-apoptotic molecules have been reported for several human cancers. How BH3-only proteins initiate the activation of Bax and Bak has still not exactly been identified and there are currently two alternative working models. In the direct activation model [56], BH3-only proteins that act as direct activators, *i.e.*, Bim and cleaved Bid (tBid), bind to Bax and Bak to trigger their activation, while BH3-only proteins that act as sensitizers, e.g. Bad, bind to the pro-survival Bcl-2 proteins. According to the indirect activation model, BH3-only proteins activate Bax and Bak indirectly by engaging anti-apoptotic Bcl-2 proteins, thereby freeing up Bax and Bak [57,58]. Furthermore, Bak activation requires inactivation of both, Bcl-X_L_ and Mcl-1 [59].

Various strategies have been developed over the last years to antagonize anti-apoptotic Bcl-2-related proteins in human cancers. For example, targeting of the protein-protein interaction site between anti-apoptotic Bcl-2 proteins and the multimeric pro-apoptotic Bcl-2 proteins Bax or Bak yielded small molecule antagonists that bind to the surface groove of Bcl-2, Bcl-X_L_ and Bcl-w in a similar manner as the BH3 domain of Bax or Bak [60]. ABT-737 represents the prototypic compound of this class of inhibitors that has been extensively characterized in preclinical models [61]. ABT-737 was shown to directly trigger apoptosis in susceptible cell lines, e.g. chronic lymphocytic leukemia cells, or to sensitize cancer cells for apoptosis [60]. Recently, ABT-737 and TRAIL were found to synergize in the induction of cell death in pancreatic cancer cells by stimulating the intrinsic and extrinsic apoptotic pathways, respectively [62]. Obatoclax, another BH3 mimetic, antagonizes Bcl-2, Bcl-X_L_, Bcl-w as well as Mcl-1 [63]. In pancreatic cancer Obatoclax has shown to potentiate TRAIL-triggered apoptosis [64]. ABT-263, an oral analogue with improved pharmacokinetic properties, is currently evaluated in early clinical trials in small-cell lung cancer and B-cell malignancies [65]. TW-37 presents another small-molecule inhibitor of Bcl-2, which was shown to inhibit cell growth and invasion and increased apoptosis in pancreatic cancer [66]. Another approach to target anti-apoptotic Bcl-2 proteins is the use of antisense oligonucleotides. For example, Bcl-X_L_ antisense oligonucleotides enhanced gemcitabine-or irradiation-induced cytotoxicity in pancreatic cancer cells [67,68].

## 5. Conclusions

Pancreatic cancer harbors multiple defects in apoptosis signaling pathways that contribute to tumorigenesis and favors treatment resistance, including high levels of anti-apoptotic proteins and/or reduced expression or function of pro-apoptotic proteins. Several components of apoptosis signaling pathways may be exploited as targets for the development of experimental cancer therapies, for example the TRAIL system, IAP proteins or anti-apoptotic Bcl-2 proteins. The transfer of this knowledge on apoptosis signaling into the design of experimental clinical trials may offer novel perspectives to improve the prognosis of pancreatic cancer patients.
